# Surgical Management of a Giant Gastric Trichobezoar in a Teenager With 17β-Hydroxysteroid Dehydrogenase 3 Deficiency: A Case Report

**DOI:** 10.7759/cureus.89913

**Published:** 2025-08-12

**Authors:** Suhaib Ahmad, Wah Yang, Sjaak Pouwels, Danielle Wilkinson, Miriam Khalil, Graham Whiteley, Ahmed Ahmed

**Affiliations:** 1 Department of General Surgery, Ysbyty Gwynedd Hospital, Bangor, GBR; 2 Department of Surgery, The First Affiliated Hospital of Jinan University, Guangzhou, CHN; 3 Intensive Care Medicine, Elisabeth-Tweesteden Hospital, Tilburg, NLD; 4 General Surgery, Betsi Cadwaladr University Health Board, Bangor, GBR; 5 Surgery, St James’s University Hospital, Leeds, GBR; 6 General Surgery, Imperial College London, London, GBR

**Keywords:** 17-beta-hydroxysteroid dehydrogenase 3 deficiency, disorder of sex development (dsd), laparoscopic gastrotomy, rapunzel syndrome, trichobezoar

## Abstract

Trichobezoars are rare gastric masses composed of ingested hair, most commonly observed in adolescents with underlying psychiatric conditions. Diagnosis is often delayed due to non-specific gastrointestinal symptoms and the patient's reluctance to disclose trichophagia. Large bezoars may extend into the small intestine and typically require surgical intervention. We report the case of a teenager with a disorder of sex development (DSD) who presented with progressive abdominal pain, persistent vomiting, and significant weight loss. Endoscopy revealed a large gastric trichobezoar extending beyond the pylorus. The patient had a known history of trichophagia and was under psychiatric care. Following post-endoscopy haemodynamic instability, she underwent emergency surgery. The trichobezoar was successfully removed via a laparoscopic-assisted mini-laparotomy, and the gastrotomy was closed in two layers. Her postoperative course was uneventful, and she was discharged with appropriate psychiatric follow-up. This case highlights the importance of early clinical suspicion in patients with persistent gastrointestinal symptoms and psychiatric comorbidities. Surgical extraction remains the treatment of choice for large bezoars, and multidisciplinary care is essential to optimise recovery and prevent recurrence.

## Introduction

Trichobezoars are rare gastrointestinal masses composed of ingested hair, typically affecting adolescent females with underlying psychiatric conditions such as trichotillomania (compulsive hair pulling) and trichophagia (hair eating) [1]. These hair masses resist both peristalsis and digestion, leading to gradual accumulation and compaction within the stomach. When the trichobezoar extends beyond the pylorus into the small intestine, the condition is termed Rapunzel syndrome.

Clinical presentation is often delayed due to vague gastrointestinal symptoms such as abdominal pain, early satiety, nausea, vomiting, and halitosis [2]. Diagnosis is further complicated in individuals with complex psychosocial or medical backgrounds, inconclusive imaging findings, or a reluctance or inability to disclose relevant behaviours.

Plain abdominal X-rays are typically non-diagnostic. Ultrasound, while non-invasive and widely accessible, is operator-dependent and may reveal a heterogeneous echogenic mass with a hypoechoic rim [3]. CT imaging is considered the gold standard for diagnosing trichobezoars, particularly when Rapunzel syndrome is suspected. It typically reveals a well-defined intraluminal mass with heterogeneous density and interspersed air bubbles, known as the "mottled gas" pattern. CT scans are also valuable for identifying complications, such as perforation or obstruction, and are instrumental in surgical planning. In this case, a CT scan was not performed due to confirmation of the diagnosis via endoscopy. However, CT imaging is generally recommended in routine practice, especially when the diagnosis is uncertain or when there is suspicion of extensive disease [4,5]. Endoscopy remains the diagnostic modality of choice, providing direct visualisation and the opportunity for therapeutic intervention; however, it is rarely effective for large bezoars due to their size and density [6].

The management of trichobezoars depends on the size, location and associated complications. Endoscopy has a role in the removal of small trichobezoars. However, for large and complicated cases, surgery is considered the definitive treatment. While open laparotomy with gastrotomy remains the standard approach, laparoscopic and hybrid techniques are increasingly being adopted in selected cases, offering advantages such as reduced postoperative pain, shorter hospital stays, and improved cosmetic outcomes. Laparoscopic approaches may be initiated but are often converted to mini-laparotomy when large bezoars cannot be safely extracted [6-8].

This report presents a complex case of a large gastric trichobezoar requiring surgical extraction. It highlights the diagnostic difficulties, surgical decision-making, and the importance of multidisciplinary care, especially when psychiatric and psychosocial factors are present.

## Case presentation

A teenager with a background of 46,XY karyotype and a confirmed diagnosis of 17-beta-hydroxysteroid dehydrogenase 3 (17β-HSD3) deficiency, a rare disorder of sex development (DSD), presented with chronic gastrointestinal symptoms. This enzymatic defect results in impaired testosterone biosynthesis and a phenotypically female appearance in genetically male individuals. The patient shared that she had noticed abnormal genital development around the age of 11 but had not disclosed this to anyone until she was diagnosed at age 13 following endocrinology referral and genetic testing. She had undergone bilateral gonadectomy and was commenced on oestrogen replacement therapy. Her care was multidisciplinary, involving paediatric endocrinology, clinical psychology, and mental health services.

Her complex medical and psychosocial background contributed to a delayed diagnosis of a rare but serious gastrointestinal condition: trichobezoar.

She presented to the emergency department with a three-year history of generalised abdominal pain, worsening over recent months. The pain was mainly in the left upper quadrant, associated with early satiety and persistent vomiting after meals for the past 2.5 months. She reported a 30 kg weight loss over 1.5 years and infrequent bowel movements every 2-3 days. A year prior, she had attended the ED with similar symptoms; an abdominal X-ray at that time was unremarkable, and she was discharged with a diagnosis of constipation.

The laboratory findings on admission with corresponding reference ranges are shown in Table 1.

**Table 1:** The laboratory findings on admission with corresponding reference ranges

Parameter	Result	Reference Range	Interpretation
White Cell Count (WCC)	20.4 ×10⁹/L	4.0 – 11.0 ×10⁹/L	Elevated (Leukocytosis)
Neutrophils	15.8 ×10⁹/L	2.0 – 7.5 ×10⁹/L	Elevated (Neutrophilia)
Platelets	630 ×10⁹/L	150 – 400 ×10⁹/L	Elevated (Thrombocytosis)
Amylase	132 U/L	30 – 110 U/L	Mildly Elevated
C-Reactive Protein (CRP)	25 mg/L	<5 mg/L	Elevated
Albumin	30 g/L	35 – 50 g/L	Low (Hypoalbuminemia)
Sodium	132 mmol/L	135 – 145 mmol/L	Mild Hyponatremia
Helicobacter pylori IgG	Negative	Negative	Normal
Anti-tTG (Tissue Transglutaminase IgA)	Negative	Negative	Normal

Abdominal ultrasound revealed a distended stomach with echogenic material and a hypoechoic band suggestive of either muscular thickening or fluid. IV fluids, antiemetics, and empirical antibiotics were started. On day 4 of admission, she disclosed a long-standing history of trichophagia, which she reported had stopped since starting fluoxetine.

**Figure 1:**
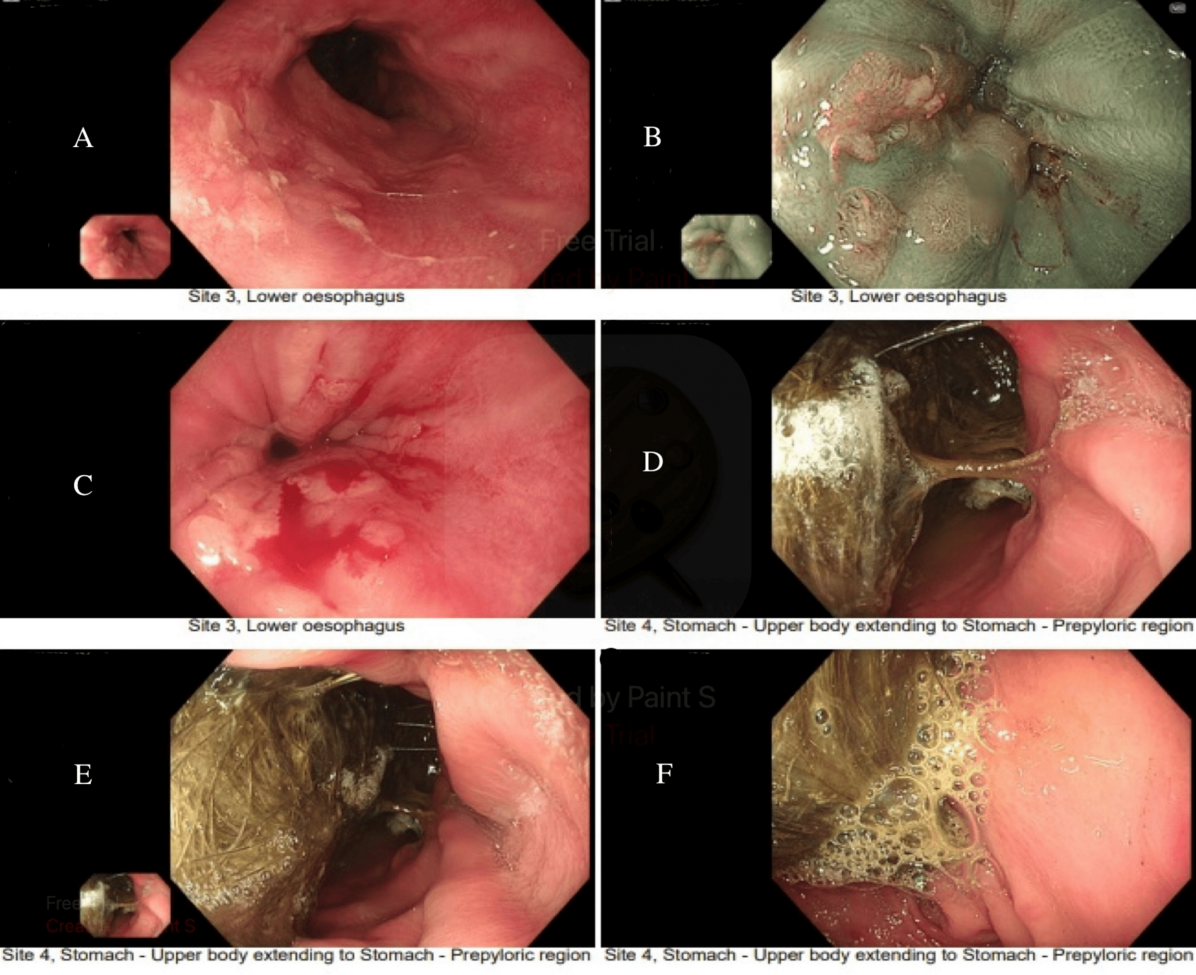
Endoscopy A: Lower Oesophagus; B: Gastroesophageal Junction (GEJ) Narrow Band Imaging (NBI); C: Gastroesophageal Junction (GEJ); D and E: Gastric Body; F: Prepyloric Region

On day 8, OGD showed a large trichobezoar (15 cm x 8 cm) occupying the stomach and extending through the pylorus (Figure 1). Post-procedure, she developed hypotension (BP 54/37), triggering a medical emergency call. Arterial blood gas (ABG) revealed pH 7.52, pCO₂ 3.35, pO₂ 6.42, and lactate 1.74. ECG showed sinus rhythm. She was resuscitated and stabilised.

**Figure 2:**
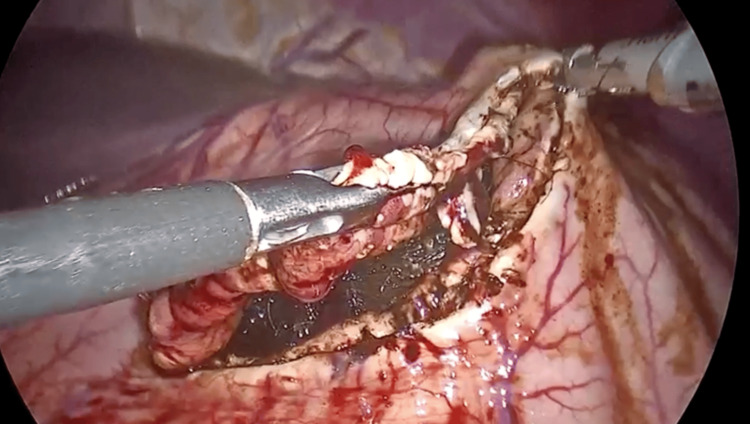
Extraction of the trichobezoar

**Figure 3:**
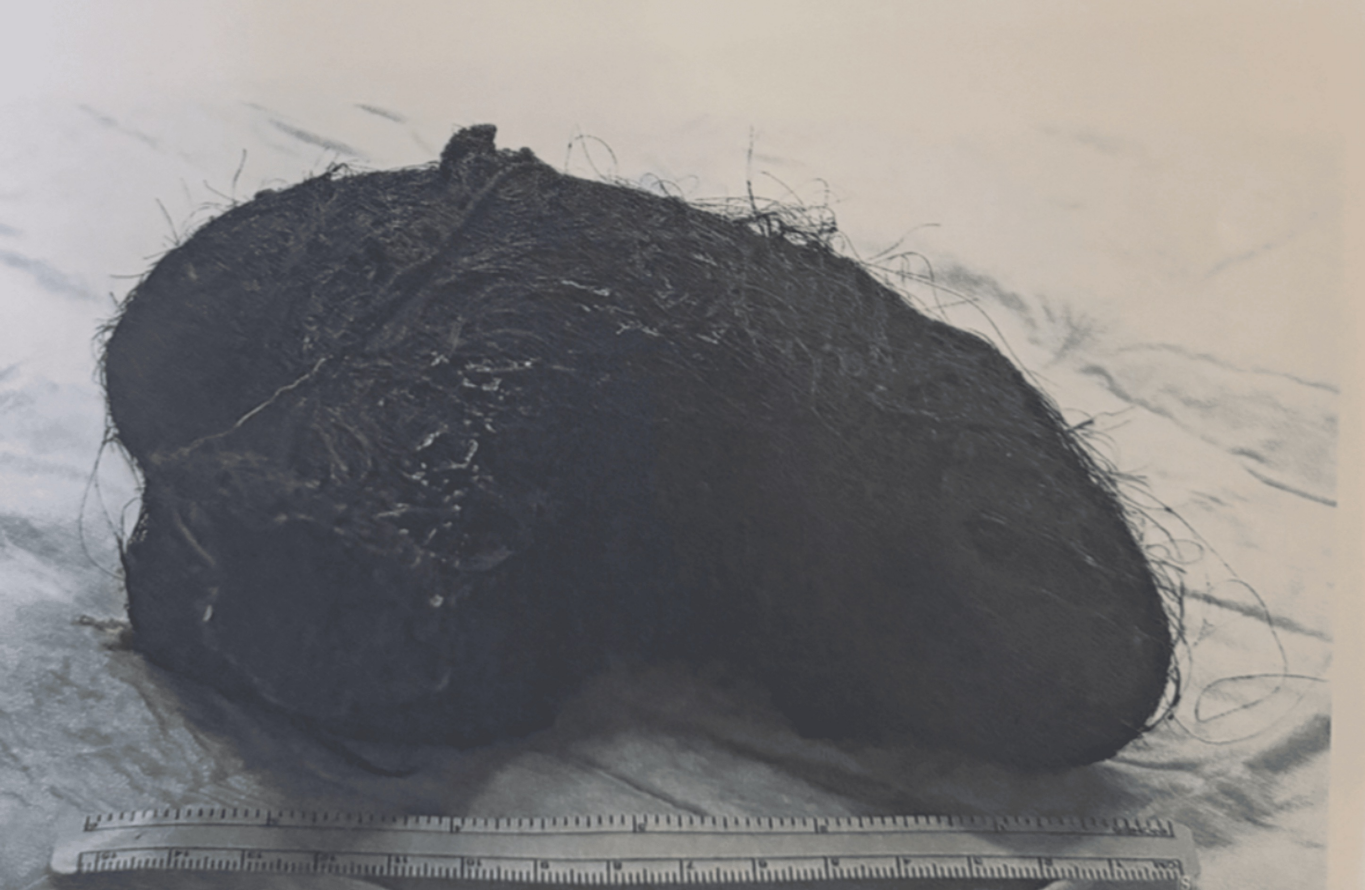
Trichobezoar

She underwent laparoscopic surgery using a 10-12 mm umbilical camera port, bilateral 5 mm subcostal working ports, and a suprapubic port. Atraumatic graspers elevated the anterior gastric wall. A 7 cm longitudinal gastrotomy was created in the gastric body using a harmonic scalpel (Figure 2). An endoscopic retrieval bag failed due to the bezoar’s size. The procedure was converted to a mini-laparotomy via extension of the suprapubic port. The mass was safely extracted using a minimal-touch technique (Figure 3). The gastrotomy was closed in two layers (inner mucosal/submucosal and outer seromuscular), both with continuous 2-0 polydioxanone suture (PDS). A 5-litre abdominal washout was performed, no contamination was noted, and a pelvic drain was left in situ (Video 1).

**Video 1:** Surgical extraction of the trichobezoar

The patient recovered uneventfully postoperatively and resumed oral intake gradually. She was reviewed by psychiatry prior to discharge and received mental health support. She was discharged in stable condition with surgical and psychiatric follow-up arranged. The patient made a full recovery following surgery and psychiatric support, highlighting the importance of multidisciplinary care.

## Discussion

This case highlights the importance of early decision-making, a flexible surgical approach, and integrated care to ensure a safe outcome and to mitigate the long-term risks in patients with large trichobezoars and underlying psychosocial conditions. Patient engagement was key in revealing the underlying behavioural disorder and supported postoperative recovery.

The approach of a laparoscopic-assisted mini-laparotomy enabled the safe, intact extraction of the bezoar. The preoperative planning with appropriate port placement was crucial due to the large size of the trichobezoar. A low midline port was considered early on, as it could be easily extended later for specimen retrieval. Furthermore, it was essential to handle the bezoar carefully to avoid breaking it apart. Fragmentation, particularly of hair, can result in peritoneal contamination, which is notoriously difficult to clear and may lead to abscesses or adhesions [9].

A longitudinal gastrotomy was performed on the anterior gastric wall using a harmonic scalpel to achieve a clean, haemostatic entry. The anterior wall, being a thick and well-vascularised part of the stomach, offers better healing potential and a reduced risk of postoperative leakage. The gastrotomy was closed in two layers, with a continuous 2-0 PDS suture for both the mucosal/submucosal and seromuscular layers, reducing leak risk and promoting healing. A 5-litre abdominal washout was performed to remove microscopic hair fragments, and a pelvic drain was placed as a precaution. These technical details align with best practice recommendations in the literature and demonstrate that thorough intraoperative execution can substantially reduce postoperative morbidity [10].

While laparoscopy offers cosmetic and recovery advantages, this case supports findings from other reports that early conversion is often warranted in large bezoars to avoid prolonged attempts, risk of perforation, or unsuccessful extraction [7,8]. In our case, this approach allowed safe removal while minimising contamination, highlighting the importance of pragmatic intraoperative decision-making.

In addition to operative considerations, this case demonstrates the role of psychiatric assessment not only in managing the immediate condition but also in preventing recurrence. The patient in our case had been previously diagnosed with depression and was treated with fluoxetine. She only disclosed a history of trichophagia after a sustained psychiatric follow-up, an important contributor to diagnostic clarification. Trichobezoars are closely associated with underlying psychiatric conditions, such as trichotillomania, trichophagia, depression, obsessive-compulsive disorder, and trauma-related disorders, which occur in approximately 13-20% of cases. This underscores the value of mental health professionals not only in treatment but in unveiling behaviours that may not be voluntarily disclosed in general medical settings [11].

In this case, the patient’s history included 17β-HSD3 deficiency (DSD) and prior gonadectomy, and this may have contributed to the psychosocial distress and secrecy. While the literature does not currently link DSDs directly with trichophagia, the burden associated with the condition, including societal prejudice, delayed diagnosis, and identity challenges, merits further investigation. The findings from this study suggest that patients with DSDs should be regarded as high risk for psychological behaviours that may impact gastrointestinal and nutritional health.

From a clinical point of view, this case underscores the importance of maintaining vigilant clinical suspicion in patients with chronic gastrointestinal symptoms, especially when accompanied by psychiatric vulnerabilities. Furthermore, this case also highlights the importance of coordinated input from mental health professionals, endocrinologists, and regular follow-up with both paediatric and surgical teams. Nutritional support should also be considered, particularly in patients experiencing prolonged vomiting and significant weight loss. An early and accurate diagnosis, followed by complete surgical removal, typically results in an excellent prognosis. When performed with a proper technique, postoperative complications like wound infection or leakage are infrequent. Prompt psychiatric intervention, as provided in this case, greatly lowers the chance of recurrence.

From a clinical policy perspective, integrating mental health services within surgical teams should be considered for adolescents and complex care units. We emphasise the need for further research on the relationship between DSDs and compulsive behaviours, such as trichophagia, as well as on strategies to promote early disclosure and timely diagnosis in this population.

Eventually, this case contributes to the evolving best clinical practice, showing how integrated care, involving both technical and psychological, can optimise outcomes in rare presentations. It also improves the psychosocial insight when managing patients with surgical pathologies.

## Conclusions

This case serves as a reminder that rare conditions, such as trichobezoar, can present with vague gastrointestinal symptoms, often attributed to more common conditions or obscured by psychosocial factors. In this patient, the delayed diagnosis resulted in significant weight loss and prolonged morbidity. The smooth recovery was only achieved through meticulous operative planning and technique, whilst psychiatric follow-up addressed the underlying behaviour, aiming to reduce the risk of recurrence. This case highlights the importance of maintaining early suspicion and intervening early once the diagnosis is established. Integrated care pathways are required for those with complex conditions, where coordinated care between surgeons, psychiatrists, endocrinologists, and nutritional teams can ensure both physical and psychological well-being.
